# In Vitro Neuroprotection of Rat Hippocampal Neurons by Manninotriose and Astragaloside IV Against Corticosterone-Induced Toxicity

**DOI:** 10.3390/molecules23123339

**Published:** 2018-12-17

**Authors:** Jing Zheng, Fang Yin, Guoqin Jin, Xueli Zhang, Lina Zhang, Zhangbin Gong, Xiangping Kang, Haiyan Hu

**Affiliations:** Department of Biochemistry, School of Basic Medical Sciences, Shanghai University of Traditional Chinese Medicine, Shanghai 201203, China; zhengjing1021@shutcm.edu.cn (J.Z.); yinfang55550@163.com (F.Y.); zhangxueli8403@163.com (X.Z.); zln_1250@163.com (L.Z.); zhangbingong@126.com (Z.G.); kxp5025@163.com (X.K.); happyan88@sina.com (H.H.)

**Keywords:** corticosterone, Manninotriose, Astragaloside IV, learning and memory relevant genes, DNA methylation

## Abstract

A chronically elevated glucocorticoid level impairs memory and cognition. Manninotriose is the main oligosaccharide of Prepared Radix Rehmanniae, and Astragaloside IV (AS-IV) is the primary ingredient of Astragali Radix; they have been reported to possess neuroprotective effects. The aim of the present study was to investigate the protective effects of Manninotriose and AS-IV on corticosterone (CORT) induced neurotoxicity and the underlying mechanisms. Primary cultured hippocampal neurons from newborn Sprague Dawley rats were treated with CORT in the absence or presence of Manninotriose and AS-IV. Cell Counting Kit-8 experiments and fluorescein diacetate (FDA)/propidium iodide (PI) double staining were conducted to assess the activity and survival rate of neurons. Quantitative Real-time PCR (qRT-PCR) and western blot analysis were performed to detect the expression of glucocorticoid receptor (GR), zinc finger protein (Zif268) and synapsin 1 (SYN1). DNA methylation of the gene promoter was assessed by bisulfite sequencing (BSP) analysis. The results demonstrated that pre-treatment with Manninotriose and AS-IV significantly improved cell viability and survival rate, and ameliorated the downregulation of GR, Zif268 and SYN1 genes in CORT injured neurons. BSP analysis revealed that CORT was able to improve the CpG island methylation rate of SYN1. AS-IV was observed to decrease the hypermethylation of the SYN1 gene induced by CORT. The results of the present study indicated that Manninotriose and AS-IV may have a protective effect against CORT-induced damage and the downregulation of learning and memory associated genes in hippocampal neurons. Regulation of DNA methylation may be important in the pharmaceutical activities of AS-IV. Thus, Manninotriose and AS-IV may be effective agents against learning and memory impairment.

## 1. Introduction

Memory disorder is a common phenomenon in the elderly, and is known as age associated memory impairment (AAMI). Not only does AAMI affect the quality of life of the elderly, but it is also an economic burden to society. It is important to understand the factors underlying the decline in memory and cognition that occurs with normal aging, not just those that result from neurodegenerative disorders. 

The hippocampus, a region in the medial temporal lobe which serves a key role in learning and memory, is an important target area for the study of AAMI [1]. Memory formation requires gene expression, which within the hippocampus has long been recognized to have a key role in synaptic plasticity and the long term retention of learned behavior [2]. Zif268, an immediate early gene, encodes a zinc finger transcription factor that regulates the expression of memory-associated proteins, and is required for the maintenance of late LTP and for the establishment of long-term memory [3]. SYN1, a member of the synaptophysin family, can regulate neurotransmitter release. Decreased expression of SYN1 results in decreased synaptic vesicle transport capacity, and consequently, the transmission, processing and storage of information is affected. It has been reported that the expression of Zif268, SYN1 and other key genes in the hippocampus decreases with aging, which is closely associated with a decline in cognitive function [4,5].

Glucocorticoid (GC) is synthesized by the adrenal cortex and has a wide range of physiological functions. GC receptor GRs are abundantly expressed in the hippocampus [6]. Previous studies have demonstrated that glucocorticoids are chronically elevated in the aged population. Sustained exposure to elevated GC is closely associated with hippocampal injury and learning and memory decline in aging [7]. Upon binding to ligand, GR becomes activated and translocates into the nucleus where it controls specific transcriptional programs and modulates gene expression. 

The mechanisms that underlie age-associated changes in gene transcription are not fully understood. Previous studies over the past few years have revealed that epigenetic modifications of gene transcription including DNA methylation serve important roles in the process of learning and memory [8,9,10]. There is a significant association between aging of the human brain and methylation of the whole genome as well as specific genes associated with synaptic plasticity and memory formation [11].

Prepared Radix Rehmanniae and Astragali Radixare commonly used in traditional Chinese medicine; previous research has indicated that they can promote learning and memory. Manninotriose is the main oligosaccharide of prepared Radix Rehmanniae. In our previous study, the results revealed that Manninotriose protected hippocampal neurons from high-concentration CORT-induced injury [12]. AS-IV extracted from the dried root of Astragali Radix is also a well-known traditional Chinese medicine that is used for the treatment of neurodegenerative diseases. A previous study demonstrated that AS-IV treatment resulted in significant axonal regeneration, reconstruction of neuronal synapses [13], and prevention of Aβ-induced neuronal death [14]. In addition, AS-IV treatment significantly attenuated 6-hydroxydopamine-induced loss of dopaminergic neurons [15]. 

In the present study, primary cultured hippocampal neurons were employed to investigate the neuroprotective effects of Manninotriose and AS-IV on CORT-induced injury and the potential underlying mechanisms. The results demonstrated that Manninotriose and AS-IV partly reversed the CORT-induced damage of hippocampal neurons. AS-IV may increase the expression of genes by inhibiting CORT-induced hypermethylation.

## 2. Results

### 2.1. Effect of Manninotriose and AS-IV on the Viability of CORT Injured Hippocampal Neurons

**Figure 1:**
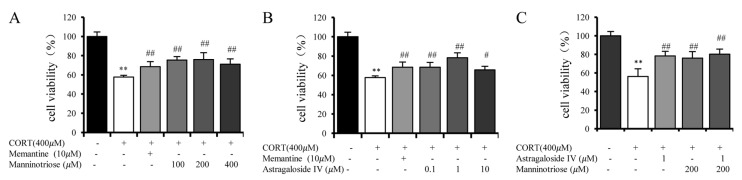
Cell viability in CORT injured hippocampal neurons pretreated with different concentrations of (**A**) Manninotriose, (**B**) AS-IV and (**C**) Manninotriose combined with AS-IV. Data are presented as the mean ± SD (*n* = 3). ** *p* < 0.05 vs. control group; # *p* < 0.05 and ## *p* < 0.01 vs. CORT group.

As shown in Figure 1, the cell viability significantly decreased by ~45% when the cultured neurons were exposed to 400 μM CORT for 24 h. Pretreatment with Manninotriose (100, 200 and 400 μM) (Figure 1A) and AS-IV (0.1, 1 and 10 μM) (Figure 1B) markedly increased the neuron viability injured by CORT. Memantine (10 μM) was used as the positive control. At the same time, combined treatment with AS-IV (1 μM) and Manninotriose (200 μM) (Figure 1C) produced a greater improvement in neuronal activity when compared with the two treatments applied alone; however, the difference was not statistically significant.

### 2.2. Effect of Manninotriose and AS-IV on the Survival of the Neurons Detected by FDA/PI Double Staining

**Figure 2:**
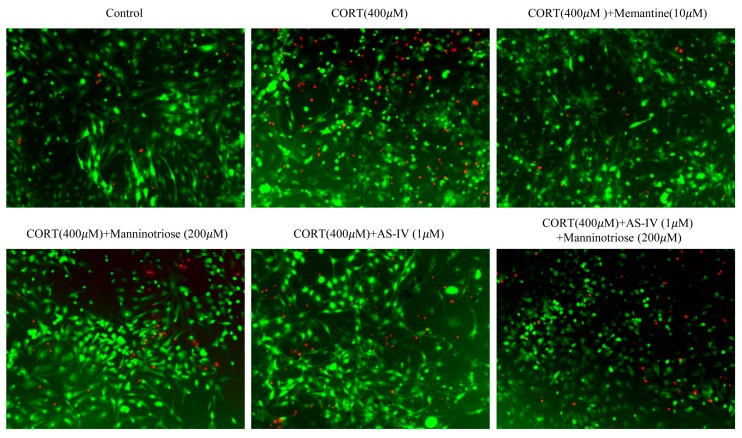
Effect of Manninotriose and AS-IV on cell survival in CORT injured hippocampal neurons as determined by FDA/PI staining. FDA (green) is taken into the living cells while PI (red) stains dead cells.

As shown in Figure 2, the number of dead neurons was markedly increased when compared with the control group following treatment with 400 μM CORT for 24 h. However, pretreatment with AS-IV (1 μM) and Manninotriose (200 μM) in the presence of CORT (400 μM) markedly reduced the level of cell death.

### 2.3. Effects of Manninotriose and AS-IV on the GR Level in CORT Injured Hippocampal Neurons

**Figure 3:**
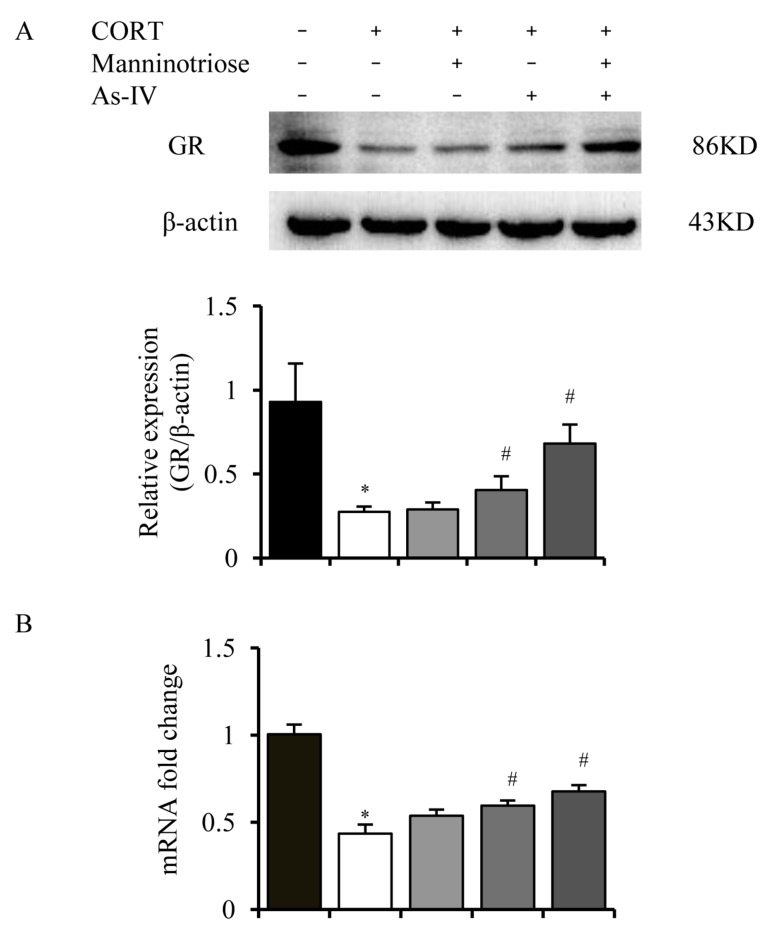
Effects of Manninotriose and AS-IV on the expression of GR in CORT treated hippocampal neurons. (**A**) GR expression assessed by western blot analysis. (**B**) GR mRNA expression in CORT-injured hippocampal neurons as determined by qRT-PCR. Data are presented as the mean ± SD (*n* = 3). ^*^ *p* < 0.05 vs. control group; ^#^ *p* < 0.05 vs. CORT group.

To determine whether GR level was altered by CORT exposure, the present study analyzed the expression of total GR protein in cultured hippocampal neurons. As shown in Figure 3, CORT significantly decreased the total GR protein level (Figure 3A) and mRNA (Figure 3B); this change was attenuated by pretreatment with AS-IV (1 μM) and combined treatment with AS-IV (1 μM) and Manninotriose (200 μM). However, the expression of GR was not significantly improved by Manninotriose (200 μM).

### 2.4. Effects of Manninotriose and AS-IV on CORT-Induced Changes in Zif268 and SYN1 Gene Expression in the Hippocampal Neurons

**Figure 4:**
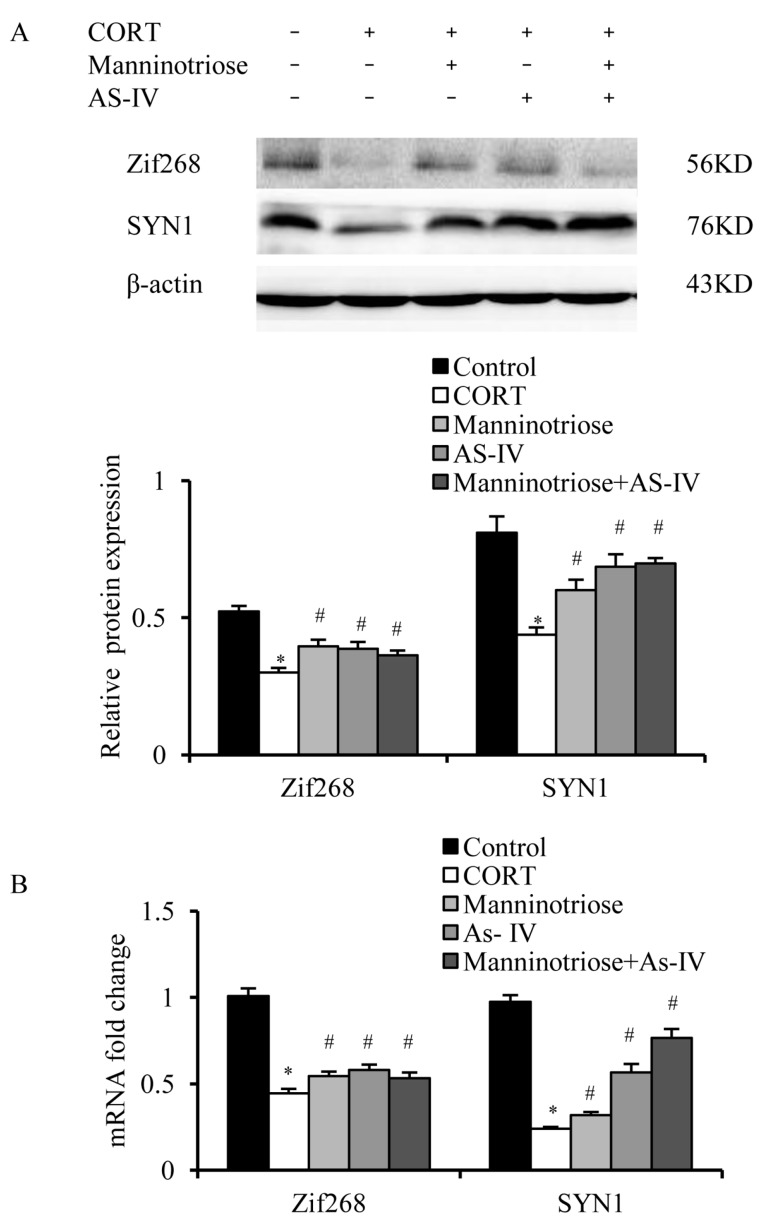
Effects of Manninotriose and AS-IV on the expression of SYN1 and Zif268 gene in CORT injured hippocampal neurons. (**A**) SYN1 and Zif268 expression assessed by western blot analysis. (**B**) Effect of Manninotriose and AS-IV on SYN1 and Zif268 mRNA expression in CORT-injured hippocampal neurons as determined by qRT-PCR. Data are presented as the mean ± SD (*n* = 3). * *p* < 0.05 vs. control group; # *p* < 0.05 vs. CORT group.

Zif268 and SYN1 protein and mRNA expression were analyzed in the hippocampal neurons. When compared with the control group, Zif268 and SYN1 expression in the CORT group decreased markedly at the protein (Figure 4A) and mRNA levels (Figure 4B). However, in the Manninotriose and AS-IV groups, the CORT induced downregulation was ameliorated (Figure 4).

### 2.5. Effects of Manninotriose and AS-IV on CpG Island Methylation in the SYN1 Gene Promoter of CORT Injured Hippocampal Neurons

**Figure 5:**
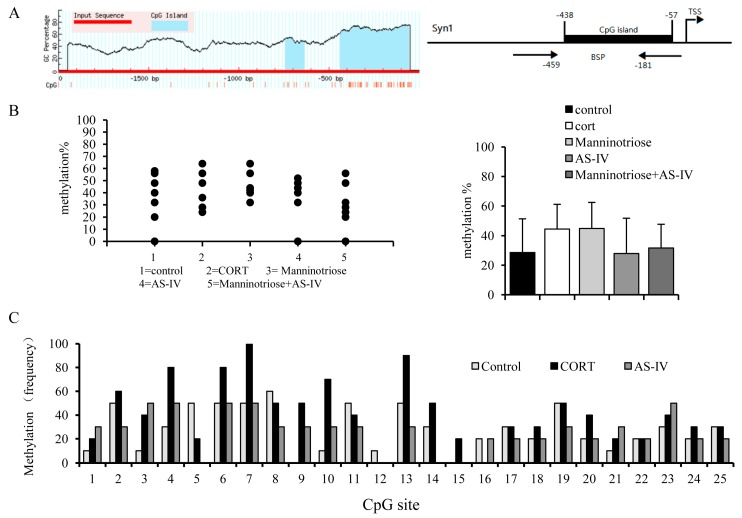
Effects of Manninotriose and AS-IV on SYN1 gene methylation in CORT injured hippocampal neurons. (**A**) The genomic structure of SYN1 including the location of the CpG islands relative to the TSS. Tss, transcriptional start site; The TSS numbers indicate the nucleotide distance from the TSS. BSP indicates the location of bisulfite sequencing PCR primers. (**B**) Methylation rate of individual clones (*n* = 10) across 25 CpGs sites of the SYN1 CpG island, as determined by BSP. (**C**) Methylation analysis of individual CpG sites (*n* = 10).

DNA methylation of gene promoters is closely associated with the repression of transcription and is negatively associated with the expression of genes. Thus, the above results provide a basis for examining genomic DNA features that may contribute to the reduced mRNA levels of SYN1. To assess the association between CORT-induced changes in gene expression and methylation, the present study investigated the methylation status of the promoter regions of the SYN1gene. One CpG island was located between −438 and −57 bp, upstream of the transcription start site of the promoter of SYN1 (Figure 5A), and there were 25 CpG sites in this island. Using BSP PCR, we observed a higher methylation rate in the corticosterone group and a lower rate in the AS-IV group and AS-IV combined with Manninotriose group. (Figure 5B). The total methylation rate was 44.4% in the CORT injured group and 28.6% in the control group. The methylation rates of the AS-IV (28%) and the AS-IV combined with Manninotriose groups (31.6%) were lower than that of the CORT group. However, using Kruskal-Wallis H test, no statistically significant total difference was observed between groups (*p* = 0.64). Under such a premise, the comparison between groups is unnecessary. 

While analyzing the overall methylation rate, we focused on methylation of the individual CpG sites. After CORT treatment, some CpG sites showed higher methylation frequencies than other sites, and the intervention effect of AS-IV was more obvious at these loci (Figure 5C). 

## 3. Discussion

Zif268 and SYN1 are required for synaptic plasticity and memory formation. Previous studies have revealed that Zif268 and SYN1 expression declines with aging, which is closely associated with the decline of cognitive function [4,5]. GR mediates the biological effects of GC by acting as a transcription factor. Upon binding to ligand, GR becomes activated and translocates into the nucleus where it controls specific transcriptional programs and modulates the expression of a variety of different genes associated with physiological responses. Sustained exposure to elevated GC is closely associated with hippocampal injury, and a decline in learning and memory in aging [7,16]. In the present study, primary cultured hippocampal neurons were treated with CORT to further explore the association between increased GC, and learning and memory decline.

In agreement with previous studies, sustained exposure to elevated GC leads to the alteration in gene expression. The present study demonstrated that the expression of Zif268 and SYN1 significantly decreased following CORT treatment. These results suggested that exposure to elevated GC may be associated with aging, and in turn learning and memory impairments via the downregulation of Zif268 and SYN1 expression. 

Numerous studies have demonstrated that DNA methylation serves a very important role in learning and memory [17,18]. The degree of DNA methylation in the brain increases during aging [11,19], and the methylation modifications of synaptic plasticity and memory formation-associated genes are also altered. SYN1 is a key neuronal phosphoprotein that drives the formation, maintenance, and rearrangement of synaptic contacts in neural circuits. Basal SYN1 gene expression is modulated by CpG methylation [20]. Haberman et al. [21] using methyl-specific PCR detected that DNA methylation in CpG island of the promoter region of SYN1 increased in a rat model of cognitive aging relative to the young. In the present study, we found that the methylation rate in the promoter region of SYN1 gene containing 25 CpG sites was increased following CORT treatment. Although there was no statistically significant difference in overall methylation rate, some CpG sites showed higher methylation frequencies than other sites after CORT treatment. Methylation of some CpG sites may play a key role in regulating the expression of SYN1. We speculate that corticosterone may affect the methylation of certain important CpG sites, thereby affecting the expression of SYN1 gene. Another in vivo study found that in the presence of high concentration of CORT, the epigenetic changes in BDNF and GDNF were enhanced and resulted in an compromised expression of the two genes in a parkinsonian rat model [22]. Chronic corticosterone exposure was also reported, which could increase expression and decrease DNA methylation of Fkbp5 in C57BL/6J mice [23]. Thus, the regulation of glucocorticoid on gene expression may be related to DNA methylation. Additional studies will be required to determine whether there is a relationship between DNA methylation changes and behavior.

The transcription factor Zif268 normally binds to the sequence near to the transcription start site (TSS) of the target gene and activates gene transcription [24]; SYN1 is one of its target genes [25]. In the present study, using a transcription factor database, the Zif268 binding sequence was identified upstream of the SYN1 TSS, and high level of CORT could cause hypermethylation of CpG sites within this binding sequence (CpG site 10 to site 14), while the methylation of DNA would inhibit the binding of transcription factors and thereby result in transcriptional silencing. Therefore, methylation may hinder the binding of Zif268 to the promoter region of SYN1, thereby affecting the transcription of SYN1. This may be one of the reasons for the decrease in SYN1 in CORT injured hippocampal neurons. In addition, Zif268 protein expression was also decreased following CORT treatment, while a decrease in Zif268 expression will directly lead to the decreased expression of the target gene.

However, does CORT reduce the expression of Zif268 and SYN1 through the same mechanism? In the present study, we did not explore this problem, which is a limitation of this study. It has been demonstrated that DNA methylation can dynamically regulate the gene transcription required for synaptic plasticity and memory processes. Zif268 is one of the genes affected by the aging process. In fact, Penner et al. [26] reported that the change in DNA methylation in the promoter region of Zif268 may possibly contribute to the changes associated with aging. Whether the age-associated hypermethylation of Zif268 is associated with elevated GC remains to be determined.

Rehmannia and Astragalus, commonly used in traditional Chinese medicine, can promote learning and memory. The present study revealed that Manninotriose and AS-IV can improve the activity and reduce the death rate of hippocampal neurons with CORT-induced injury. It was also observed that simultaneously using the two drugs generated a better protective effect than when used alone, even though the difference was not statistically significant. The change in efficacy following treatment combination suggested that there may be interactions between the two components, either based on the mechanism of action or on physicochemical properties; this requires further pharmacological studies to elucidate the mechanism further. A number of studies have indicated that prolonged GC exposure could be neurotoxic and may cause neuronal death in the hippocampus mainly by affecting neuronal energetics, increasing glutamate accumulation, or increasing the free cytosolic calcium load and oxygen radical accumulation [27]. The prominent antioxidant effect [28] and improvement in intracellular calcium handling via Ca-ATPase [29] may contribute to the anti-CORT neurotoxicity of AS-IV. To the best of our knowledge, the neuroprotective effect of Manninotriose has not been reported in previous studies. The present studies provide some insight into the neuroprotective effects of Manninotriose, however, the underlying mechanism requires further investigation.

The expression of SYN1, Zif268 in injured neurons was significantly raised when Manninotriose and AS-IV were used alone or in combination, and the improvements observed following the combination of the two components was slightly better than those of the single use treatments. As a transcription factor, GR binds to specific response elements and regulates the transcription of the target gene. When the GC level is continuously increased, GR expression decreases, and the expression of the target genes, which serve an important role in learning and memory, are also affected. The results of the present study indicated that the upregulation of SYN1and Zif268 induced by AS-IV not Manninotriose may be associated with increased GR expression. BSP sequencing analysis revealed that AS-IV had a certain degree of regulation on CpG methylation of the SYN1 gene in CORT injured neurons, especially on CpG sites within the binding sequence of Zif268; however, Manninotriose had no effect on this. This result indicated that the regulation of gene expression by AS-IV might be related to DNA methylation. DNA methylation might not be the main mechanism regulating the expression of SYN1 by Manninotriose. In the present study, we did not study the underlying mechanism of the action of Manninotriose from other aspects, which is a limitation of this study. Histone deacetylation could represent an additional epigenetic mechanism, in addition to DNA methylation, to induce gene repression. Our future experiments will aim at better characterizing SYN1 promoter epigenetic marks under high GC level condition and the effect of Mannotriose.

In conclusion, the results of the present study revealed that Manninotriose and AS-IV can protect neurons from neurotoxicity induced by CORT in vitro. To the best of our knowledge, for the first time the neuroprotective effect of AS-IV was observed to be associated with the regulation of DNA methylation disturbed by CORT. Further studies are required to determine the neuroprotective effects of these two compounds in vivo. Further experiments are also required to determine the role of CORT and AS-IV in regulating DNA methylation.

## 4. Materials and Methods

### 4.1. Chemical Compounds

AS-IV was purchased from Shanghai Seebio Biotech, Inc. (Shanghai, China). CORT was purchased from Sigma-Aldrich (St. Louis, MO, USA). Manninotriose was purchased from Shanghai Tanto Biotech, Co., Ltd. (Shanghai, China). Memantine was purchased from LKT Laboratories, Inc. (St. Paul, MN, USA).

### 4.2. Primary Culture of Hippocampal Neurons

Primary hippocampal neurons were prepared from newborn Sprague Dawley rat, less than 24 h old, described previously [30] with some modifications. After anesthesia and sterilization, the hippocampus was dissected out and cut into small pieces, followed by 20 min digestion in 0.125% trypsin (Thermo Fisher Scientific, Waltham, MA, USA) at 37 °C. 10% fetal bovine serum (FBS) (Thermo Fisher Scientific, Waltham, MA, USA) was used to stop the digestion. After terminating the reaction, the digested tissue was triturated and allowed to sit undisturbed for 5 min, which allowed non-dissociated tissue to settle at the bottom. The upper fraction was removed to another tube and diluted by DMEM (Hyclone Laboratories, Inc, Logan, UT, USA) with 10% FBS to a density of approximately 500,000 cells/mL. The cell suspensions were inoculated in culture plates coated with L-poly lysine (Sigma-Aldrich, St. Louis, MO, USA) and cultured in a 37 °C/5% CO_2_ incubator. When the neurons adhered, the medium was changed to neurobasal medium (Thermo Fisher Scientific, Waltham, MA, USA). Half of the maintenance medium was replaced every 3–5 days by Neurobasal medium containing B27 supplement (Thermo Fisher Scientific, Waltham, MA, USA). The culture neurons were used for in vitro studies at day 8. The purity of our primary cultured nerve cells was approximately 90%, as judged by immunocytochemistry for glial fibrillary acidic protein and neuron-specific enolase in our previous work [12].

### 4.3. Determination of Cell Viability

Cell viability was evaluated with the Cell Counting Kit-8 (CCK-8, qcbio, Shanghai, China) according to the manufacturer’s protocol. Cells were incubated in 10% CCK-8 for 2 h at 37 °C, and the optical density value was measured at 450 nm with a microplate reader Synergy2(BioTek Instruments, Inc., Winooski, VT, USA). 

### 4.4. FDA/PI Staining

The cells cultured in 96-well plate were incubated with 100 μg/mL FDA for 5 min, followed by 1 μg/mL PI for another 5 min, and then washed twice with water. Inverted fluorescence microscopy (ZEISS, Oberkochen, Germany) was used to observe the stained cells.

### 4.5. Western Blotting

Cultured cells were lysised by RIPA buffer supplemented with complete protease inhibitor (Roche, Mannheim, Germany) and phenylmethylsulfonyl fluoride (Sigma-Aldrich, St. Louis, MO, USA). Aliquots (20 μg) of total protein extracts were resolved on SDS-PAGE gels and transferred onto PVDF membranes. The membranes were then incubated with antibodies against GR (1:10000; Cell Signaling Technology, Danvers, MA, USA), Zif268 (1:500; Abcam, Cambridge, UK), SYN1 (1:200; Abcam, Cambridge, UK), or β-actin (1:1000; Santa Cruz Biotechnology, Dallas, TX, USA) overnight at 4 °C. Subsequently, the membranes were incubated with specific HRP-conjugated secondary antibodies (1:10,000; Sigma-Aldrich, St. Louis, MO, USA) for 1 h at 37 °C Signals were detected using western blot chemiluminescence HRP substrate (Takara, Kusatsu, Shiga, Japan). Image J software was used to analyze the result.

### 4.6. RNA Extraction and qRT-PCR

Total RNA was extracted from the cultured cells using Trizol Reagent (Thermo Fisher Scientific, Waltham, MA, USA) according to the manufacturer specifications. Reverse transcription was carried out using ReverTra Ace qPCR RT Master Mix (TOYOBO, Osaka, Japan). PCR was performed using THUNDERBIRD qPCR Mix (TOYOBO, Osaka, Japan) by the Step one PCR System (Applied Biosystems) with β-actin used as the reference gene. The PCR conditions were 1 cycle of 1 min at 95 °C and 40 cycles of 15 s at 95 °C, 45 s at 60 °C. The data were analyzed by the 2^−∆∆Ct^ method to calculate the fold-change. The primers used are depicted in Table 1.

### 4.7. DNA Isolation, Bisulfite Conversion and Methylation Analysis

DNA was isolated by the ZR Genomic DNA™-Tissue MiniPrep kit (Zymo Research, Irvine, CA, USA) using the precipitation protocol recommended by the producer. The quality and concentration were assessed using gel electrophoresis and fluorescence intensity analysis on a Synergy 2 Multi-Mode Reader (BioTek Instruments, Inc. Winooski, VT, USA). Bisulfite conversion and subsequent purification of the isolated DNA were performed using the EZ DNA Methylation–Gold Kit (Zymo Research, Irvine, CA, USA) according to the instructions. Bisulfite-treated DNA was used as the template for PCR performed by Taq Premix kit from ZYMO Research. The BSP specific primers were designed according to the location of the SYN1CpG islands. The Primer sequences are as follow F-5′GAATTTTTATTTTTATTTTTTAAATTG3′ and R-5′CCCCTATCTTACACCTCTTATAATAC3′. The PCR conditions were 95 °C for 10 min, 95 °C for 30 s, 52 °C for 30 s, and 72 °C for 30 s for 45 cycles, followed by one cycle of 72 °C for 7 min. The PCR products were purified and inserted into the pLB plasmid (Tiangen, Beijing, China), and transformed into competent Escherichia coliTOP10. Ten clones were sequenced by Biologo Company (Shanghai, China).

### 4.8. Statistical Analysis

The results are presented as the mean ± SD of at least three independent experiments, Statistical analysis was performed using one-way analysis of variance followed by Least Significant Difference test, and the DNA methylation rate was analyzed by nonparametric test Kruskal-Wallis H test by SPSS Software, ver. 16.0 (SPSS, Inc., Chicago, IL, USA) and p values less than 0.05 were considered to be statistically significant.

## Figures and Tables

**Table 1:** The primers used for Quantitative Real-time PCR (qRT-PCR)

Gene	Primers Sequence
Zif268(NM_012551.2)	Forward GCGAACAACCCTACGAGCAC Reverse GCGGCCAGTATAGGTGATGG
SYN1(NM_001110782.2)	Forward GACAACCAACATGACTTCCAGG Reverse TTCCAGTTCCCTGACACTGATG
β-actin(NM_031144.3)	Forward CCCATCTATGAGGGTTACGC Reverse TTTAATGTCACGCACGATTTC
